# Effects of Specially Designed Energy-Restricted Diet on Anthropometric Parameters and Cardiometabolic Risk in Overweight and Obese Adults: Pilot Study

**DOI:** 10.3390/nu16203453

**Published:** 2024-10-11

**Authors:** Ana Petrovic, Snezana Jovicic, Margarita Dodevska, Brizita Djordjevic, Neda Milinkovic, Nevena D. Ivanovic

**Affiliations:** 1 Nutritional Studio Ana Petrovic, Bulevar Oslobođenja 79, 11000 Belgrade, Serbia; anapetrovic.nutricionista@gmail.com; 2 Department of Medical Biochemistry, Faculty of Pharmacy, University of Belgrade, Vojvode Stepe 450, 11221 Belgrade, Serbia; snezana.jovicic@pharmacy.bg.ac.rs (S.J.); neda.milinkovic@pharmacy.bg.ac.rs (N.M.); 3 Institute of Public Health of Serbia, Dr. Milan Jovanovic Batut, Dr. Subotica 5, 11000 Belgrade, Serbia; margarita_dodevska@batut.org.rs; 4 Department of Bromatology, Faculty of Pharmacy, University of Belgrade, Vojvode Stepe 450, 11221 Belgrade, Serbia

**Keywords:** energy-restricted diet, macronutrient variation, anthropometric parameters, cardiometabolic risk, weight loss

## Abstract

Background/Aims: This study examined the effects of a specially designed energy-restricted diet with alternate carbohydrate intake on body composition and cardiometabolic risk factors in overweight and obese adults. The aim was to assess whether the intervention could lead to significant weight loss, improve body composition, and reduce cardiometabolic risks. Methods: Sixty-five participants (34 women, 31 men) with an average BMI of 31.8 ± 9.1 kg/m^2^ (women) and 34.1 ± 6.4 kg/m^2^ (men) participated in a 14-week intervention. The diet included different days of carbohydrate intake and a 20% reduction in total daily energy consumption. Anthropometric measurements and biochemical parameters, including predictive indices of cardiometabolic risk, were determined at baseline and after the intervention. Results: The intervention resulted in a significant reduction in body weight (mean weight loss of 17%, *p* < 0.001), with 64.6% of participants achieving a weight loss of at least 10%. Muscle mass as a percentage of total body weight increased. Cardiometabolic improvements were observed in fasting blood glucose (from 5.4 to 4.9 mmol/L, *p* < 0.001) and LDL cholesterol (from 3.38 to 2.81 mmol/L, *p* < 0.001). Gender-specific differences were found, particularly in HDL-C, which decreased significantly in women (*p* = 0.013), while there was a non-significant increase in men. Cardiometabolic indices, including the Visceral Adiposity Index (VAI) and the Cardiometabolic Index (CMI), also improved significantly. Conclusions: The alternate carbohydrate diet improved body composition, cardiometabolic health, and treatment adherence through metabolic flexibility. However, the short duration of this study and the lack of a control group suggest that further research is needed to assess long-term sustainability.

## 1. Introduction

Obesity is a complex, multifactorial condition defined as excessive fat accumulation that poses significant health risks, including increased susceptibility to cardiometabolic diseases such as type 2 diabetes, hypertension, and dyslipidemia [1]. The rate of obesity continues to increase globally, and it has more than doubled in adults since 1990, reaching a rate of 43% of adults who are overweight or obese in 2022 [2]. Unhealthy lifestyles, especially improper dietary habits besides physical inactivity, are major contributors to the development of obesity. Therefore, lifestyle advice, including an individual-adapted diet, remains a cornerstone for obesity treatment [1,2]. Consequently, alongside the increase in obesity incidence, the demand for effective dietary strategies that prevent weight gain, induce weight loss, and reduce the risk of chronic disease also increased [3]. An energy-restricted diet, usually targeting 20–30% of the estimated energy requirements of overweight or obese individuals, is one of the most commonly employed strategies for reducing body weight (BW) and body fat (BF) content [4,5]. The literature data confirmed the positive influence of energy-restricted diets on cardiometabolic risk factors, such as blood pressure, dyslipidemia, and glucose intolerance, which often improve after only a 5% loss of initial BW [6]. However, besides calorie restriction, manipulation in the nutrient composition of diet also has an influence on the process of weight loss and cardiometabolic risk factors, mostly relying on the well-established facts that alterations in macronutrient composition can influence endocrine balance and the gut microbiome, potentially affecting fat storage [1,7]. Several types of energy-restricted diets with altered macronutrient compositions have been examined for their effectiveness in promoting weight loss and improving cardiometabolic health [1,8]. Numerous studies have confirmed that carbohydrate-restricted diets positively impact weight loss and body composition, as well as improvements in blood glucose levels and lipid status, which are well-recognized cardiometabolic risk factors [9]. Low or moderate carbohydrate intake (less than 26% or 45% of overall energy intake, respectively) is often replaced with higher fat and protein consumption, which suppresses appetite by slowing gastric emptying and promoting prolonged satiety [10]. Namely, a high protein diet in which >20% of energy is derived is associated with greater satiety, reduced appetite, and preservation of lean body mass during reduced-calorie, low-fat diets [11,12]. Moreover, isocaloric diets that are lower in carbohydrates but higher in fat reduce insulin secretion since dietary fat does not stimulate insulin secretion. This reduction promotes fat loss from adipose tissue and makes free fatty acids available for use by metabolically active tissues [11]. Studies have shown that switching the diet from high carbohydrate to high fat (or vice versa) can impair the body’s ability to switch efficiently between glucose and fatty acid oxidation. This adaptability is critical for maintaining insulin sensitivity and metabolic health [13,14]. In addition, different macronutrient compositions, such as low-carbohydrate or high-fat diets, can significantly affect metabolic pathways and the body’s ability to utilize different fuel sources. This is particularly important in conditions such as obesity and type 2 diabetes, where metabolic inflexibility is common. Data from the literature also suggest that metabolic flexibility is associated with changes in the metabolism of muscle and adipose tissue, which are influenced by diet and other factors such as exercise [14]. However, energy-restricted diets may lead to adaptive thermogenesis, in which energy expenditure decreases more than would be expected from weight loss alone, hindering the sustainability of weight loss and promoting weight regain [15]. Data from the literature also suggest that strategic periods of higher calorie intake or intermittent fasting may prevent the metabolic adaptation typically observed with continuous energy restriction. This emphasizes the importance of alternating energy restriction and macronutrient metabolism to maintain fat loss and, thus, improve long-term weight loss outcomes [16]. Nonetheless, adherence to these energy-restricted and macronutrient-altered diets is a critical factor in their success [17]. Compliance tends to decrease over time due to various factors, such as biological, psychological, and socio-environmental factors related to eating, energy, and satiety [18]. Behavioral counseling to support lifestyle changes that counteract metabolic adaptations to weight loss shows promise for enhancing the success of weight loss interventions. For example, the inclusion of days off, a common practice in intermittent dieting, provided psychological flexibility and relief from constant restriction, which has been shown to increase adherence by reducing feelings of deprivation and promoting long-term adherence to the overall diet plan [4]. In addition, interventions that focus specifically on dietary factors such as protein and fiber can increase metabolic rate and offset the slight decrease in metabolism that accompanies weight loss [19]. One of the most effective strategies to increase adherence is regular counseling sessions, which significantly increase motivation to lose weight [20]. On the other hand, according to the study conducted by Trujillo-Garrido et al., one of the most common barriers to diet adherence was that patients were not prescribed a diet [20].

Anthropometric parameters are crucial in predicting cardiometabolic risk. While Body Mass Index (BMI) has traditionally been used as a primary measure, other parameters that are better indicators of general or central obesity have been shown to be superior in predicting cardiometabolic risk, such as waist circumference (WC), waist-to-hip ratio (WHR), and waist-to-height ratio (WtHR), and complement the use of BMI in identifying cardiovascular risk factors [21,22]. Moreover, besides anthropometric parameters, biochemical parameters should be considered for predicting cardiometabolic risk. Therefore, other predictive indexes for metabolic syndrome (MetS) have been developed, integrating both anthropometric parameters and biochemical factors as markers of adiposity [23,24].

The main aim of this study was to investigate the effects of an energy-restricted diet with alternating high, medium, and low carbohydrate intake on anthropometric parameters, biochemical markers, and cardiometabolic risk factors in overweight and obese adults. This intervention was designed to promote weight loss, improve body composition, and reduce cardiometabolic risks. Namely, the inclusion of anthropometric parameters (such as waist circumference, waist-to-hip ratio, and waist-to-height ratio) and biochemical markers (such as lipid levels and glucose) is essential for understanding the overall effect of our intervention on cardiometabolic health. These measurements were crucial to assessing how the alternative carbohydrate diet could improve both body composition and cardiometabolic risk. By examining both anthropometric and biochemical indices, this study aimed to provide a more comprehensive understanding of how the intervention could reduce cardiometabolic risk in overweight and obese individuals. This study also aimed to investigate the differential effects of the intervention on male and female participants and to assess whether gender-specific responses were observed in terms of fat loss, metabolic flexibility, and cardiometabolic improvements. To encourage participation in this study, regular counseling sessions were offered to personally guide and motivate participants throughout the duration of this intervention. We hypothesized that the diet intervention combined with structured counseling and days off would lead to improved diet adherence, significant reductions in body weight and fat mass, and improvements in anthropometric and biochemical markers of cardiometabolic health.

## 2. Materials and Methods

### 2.1. Subjects

Participants were recruited between August 2023 and December 2023 via social media platforms (Instagram, Facebook) and flyers. Participants were screened for eligibility according to the inclusion criteria during informational sessions held at the local accredited nutrition studio (Belgrade, Serbia), where the entire clinical trial was conducted. Participants were eligible if they were ≥18 years old, had a body mass index (BMI) of ≥25 kg/m^2^, and had not participated in a diet or exercise program or lost weight in the last six months. Exclusion criteria were energy expenditure through physical activity of more than 1000 kcal/week, current use of prescription medications for hyperglycemia, hyperlipidemia, hypertension, diabetes, thyroid disease, androgenic problems, or weight loss (such as Ozempic), use of dietary supplements known to affect lipid and glucose profile or muscle mass, and presence of celiac disease or other intestinal disorders.

The research protocol was approved by the Ethics Committee of the Faculty of Pharmacy, University of Belgrade, Serbia (Code of Ethics Committee Approval: 858/2). This study was conducted in accordance with the Declaration of Helsinki and the 2005 Additional Protocol to the European Convention for the Protection of Human Rights and Medicine on Biomedical Research. Before the start of this experiment, all participants were informed in detail about the aims of this study, possible risks, and benefits. They were also informed of their right to withdraw from this study at any time. All participants gave their written consent to participate and for the anonymous use of their biochemical and anthropometric data for further scientific research.

**Figure 1:**
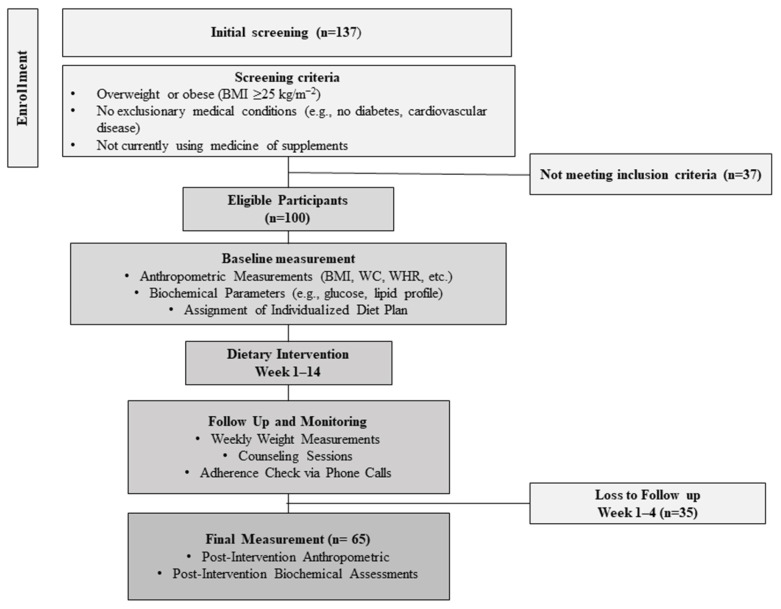
Flowchart of participant enrollment and dietary intervention process.

A total of 100 individuals of both sexes met the inclusion criteria, completed the baseline tests, and began participating in this diet program. Twenty-eight participants withdrew during the first month because they did not want to continue the diet, mostly due to a lack of motivation, and seven participants were excluded because they did not follow the diet instructions. Thus, 65 participants completed the 14-week study and were included in this analysis. A flowchart of participant enrollment and the dietary intervention process is shown in Figure 1.

### 2.2. Dietary Intervention and Diet Composition

The dietary intervention lasted 14 weeks. Daily calorie consumption was approximately 20% below total daily energy expenditure (TDEE) each week, but daily calorie restriction varied by 200 kcal up to (but not beyond) an energy restriction equivalent to resting metabolic rate (RMR), mainly depending on macronutrient distribution, with greater restriction on low-carbohydrate days and less restriction on high-carbohydrate days, ensuring that energy intake was never below RMR. Energy restriction was achieved primarily through reduced carbohydrate intake, while protein intake was above standard dietary recommendations to maintain lean body mass during weight loss. Fat intake was adjusted to stay within the recommended dietary guidelines.

RMR was calculated using the Harris–Benedict formula:

Male:

RMR [kcal/24 h] = 66.47 + (13.7 × body weight [kg]) + (5 × height [cm]) − (6.8 × age [years])

Female:

RMR [kcal/24 h] = 65.51 + (9.6 × body weight [kg]) + (1.8 × height [cm]) − (4.7 × age [years])

The TDEE was calculated by multiplying the calculated RMR value by a factor for the physical activity level (PAL factor). The PAL factor was based on available indicators for adults with low physical activity (PAL = 1.4).

**Figure 2:**
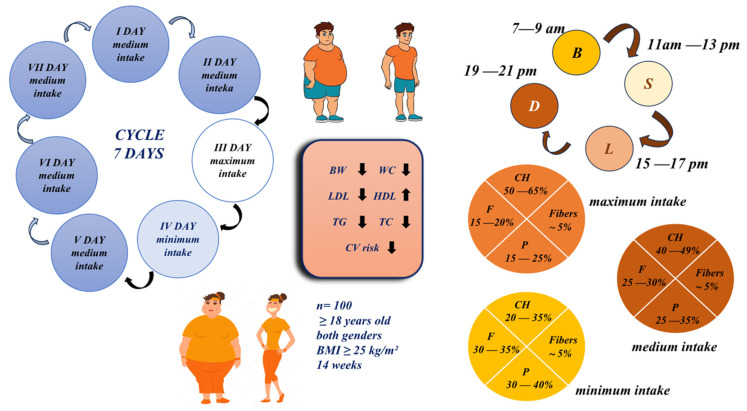
Concept of dietary intervention: weekly cycle of macronutrient intake and meal timing. BW—body weight; WC—waist circumference; TC—total cholesterol; LDL—low-density lipoprotein; HDL—high-density lipoprotein; TG—triglycerides; CV—cardiovascular; CH—carbohydrates; P—protein; F—fat; B—breakfast; L—lunch; D—dinner; S—snack.

The diet patented by Petrovic A., 2022 [25], consisted of a cycle of days in which the proportion of carbohydrates, fats, and proteins in the total energy intake alternated. For ease of identification, the days are named according to the amount of carbohydrates consumed on that day: the minimum intake day; the intermediate intake day; and the maximum intake day. The diet plan consisted mainly of days with medium carbohydrate content, with periodic fluctuations between days with maximum and minimum carbohydrate content in order to promote metabolic flexibility while ensuring overall adherence to the dietary structure. Specifically, each weekly cycle consisted of a structured sequence of seven days. Participants followed the following fixed pattern of carbohydrate intake: five consecutive days of medium carbohydrate intake, followed by one day of high carbohydrate intake, and one day of low carbohydrate intake. The intake of fats and proteins was adjusted accordingly each day. The scheme of the weekly cycle of macronutrient intake and meal timing is presentedin Figure 2.

On low-carbohydrate days, the proportion of carbohydrates in the total energy intake is 20–35%, whereby the intake of proteins and fats is increased in order to maintain the total energy content. On days with a medium carbohydrate content, carbohydrates make up 40–49% of the total energy intake, while a balanced intake of proteins and fats ensures a stable energy supply and supports the metabolism. On high carbohydrate days, carbohydrate intake is 50–65% of total energy intake to replenish glycogen stores, with fat intake reduced accordingly. On each day, regardless of the rotation of macronutrient intake, dietary fiber was supplied in an amount corresponding to approximately 5% of total energy intake. The composition of the diet is shown in Table 1. This dietary program followed a healthy eating pattern, with an emphasis on unsaturated fats from sources such as olive oil, nuts, seeds, and oily fish, while saturated fats were kept to a minimum. Carbohydrates came mainly from whole grains, legumes, and vegetables, with an emphasis on complex carbohydrates, especially fiber. Protein intake came from a combination of plant and animal sources, including lean meat, fish, and legumes, which provided sufficient protein for muscle maintenance. An example of a participant’s meal plan with macronutrient composition for one cycle according to the participant’s anthropometric characteristics can be found in Supplementary Materials (Table S1 and Table S2, respectively).

The cyclic rhythm of macronutrient intake was repeated for three months, according to the appropriate scheme. On a monthly basis, the cycles change according to the following scheme:

- days 1–21 => 3 consecutive cycles;
- day 22 => rest day, i.e., a day without the restriction of nutrient intake;
- day 23 => a day of the minimum intake;
- days 24–30 => another cycle of seven days.

In this diet, all meals were precisely planned on a weekly basis, including three main meals and one snack per day. Subjects were instructed to wait at least 4 h after finishing the previous meal before consuming the next meal. During the 14 weeks of the experiment, the participants ate their self-prepared meals according to the detailed planned recipes. Before the start of the experiment, they were informed during individual visits with experienced dietitians about which dishes were planned for each meal and given verbal and written instructions on how to prepare them. They also received a comprehensive nutrition guide with a detailed list of recommended meals, exact portion sizes for each food, step-by-step recipes for meal preparation, and a suggested weekly meal plan tailored to their preferences and nutritional needs. The meal plans were tailored to the participants’ individual characteristics and preferences to ensure that the diet was both practical and sustainable for each participant. This individualized approach was designed to maximize adherence to the diet and accommodate personal dietary restrictions or preferences.

To further support adherence to the diet, participants attended weekly counseling sessions with an individual dietitian, either on-site or online, depending on availability. These sessions were designed to encourage participants to stick to the diet, address issues, and help them achieve their weight loss goals. In addition to the weekly sessions, adherence to the diet was closely monitored through twice-weekly telephone interviews. During these calls, participants were asked if they were sticking to the diet plan and if they were experiencing any difficulties. This close monitoring of adherence was maintained throughout this study to ensure that participants were following the prescribed diet plan as closely as possible. Participants were also advised to engage in moderate physical activity (walking, cycling, swimming, etc.) for at least 30 min 5 times a week. In addition, at the end of the dietary intervention, participants were asked to complete a satisfaction questionnaire, which was used in the study by Mateo-Gallego et al. [26], to answer questions about satisfaction, health, and hunger.

### 2.3. Measurements

#### 2.3.1. Anthropometry and Body Composition

Anthropometric parameters were used as primary measurement parameters. The physical examination, medical history, and biochemical analysis of the subjects were documented before the start of this diet program (T0) and at the end of the intervention (T1) between 6.00 and 10.00 a.m. Subjects were instructed to come fasted. Body weight (BW) and body composition parameters (body fat percentage, skeletal muscle mass, and body water) were measured every two weeks via bioelectrical impedance through bipolar 8-Sensor technology using both hands and feet for an entire body measurement (Tanita BF511, Omron Corporation^®^, Tokyo, Japan). Height at the vertex was measured to the nearest 0.5 cm using a stadiometer (Seca 284, Hamburg, Germany). BMI was calculated as weight (kg) divided by height (m) squared. Waist circumference (WC) was at the narrowest part of the waist, between the lowest rib and the top of the iliac crest, using a flexible, non-elastic, calibrated tape measure. Hip circumference (HC) was measured at the thickest part of the buttocks. All measurements before and during this study were taken by the same operator, trained dietitians, using standardized procedures to ensure accuracy and consistency [27,28].

The following indices were calculated according to the formulas of Lazzer et al. [18]:

Waist-to-Hip Ratio (WHR): WC (cm)/HC (cm);

Waist-to-Height Ratio (WtHR): WC (cm)/height (cm);

Body Mass Fat Index (BMFI): BMI (kg m^−2^) × FM (%) × WC (m);

Cardiometabolic Index (CMI): WtHR × TG (mmol L^−1^)/HDL-C (mmol L^−1^).

The following mathematical formulas were used to calculate the gender-specific values of the obesity indices [22]: 

Visceral Adiposity Index (VAI) (males): [WC/(39.68 + 1.88 × BMI)] × (total TG/1.03) × (1.31/HDL-C);

VAI (women): [WC/(35.58 + 1.89 × BMI)] × (total TG/0.8) × (1.52/HDL-C);

Lipid Accumulation Product (LAP) (men): Total TG × (WC-65);

LAP (women): Total TG × (WC-58).

In these formulas, HDL-C and total TG levels, weight, WC and age were expressed in mmol/L, kg, cm, and years, respectively.

#### 2.3.2. Biochemical Analysis

The blood samples were taken in the morning hours (between 7:00 and 9:00 a.m.) by trained medical personnel using venipuncture. Sampling was performed after a 12 h overnight fasting period, and participants refrained from physical activity and smoking prior to the procedure. A closed system with a BD Vacutainer (Beckton Dickinson, U.K. Ltd., Oxford, UK) and a standard 22-gage needle (SWG) was used for venipuncture. A BD Vacutainer with ethylenediaminetetraacetic acid (EDTA) was used for total blood count measurement and plasma separation, while a BD Vacutainer with serum separator gel was used for serum collection. For fibrinogen and erythrocyte sedimentation rate, a BD Vacutainer with 3.2% sodium citrate as an anticoagulant was used.

Plasma samples were first stored at +4 °C and then centrifuged at 1500× *g* for ten minutes. For serum separation, the blood samples were centrifuged at 3000 rpm for ten minutes at room temperature immediately after a clotting time of approximately 45 min. Serum samples were analyzed using an Olympus AU400 biochemistry analyzer (Beckman Coulter Biomedical GmbH, Hamburg, Germany). Standardized spectrophotometric methods and commercial reagents (Beckman Coulter Biomedical GmbH, Hamburg, Germany, and BioSystem, Barcelona, Spain) were used to measure the following parameters: glucose; urea; creatinine; uric acid; total bilirubin; serum iron; aspartate aminotranspherase (AST); alanine aminotranspherase (ALT); total cholesterol; high-density lipoprotein (HDL-C); low-density lipoprotein cholesterol (LDL); triglycerides. Standardized immunoturbidimetric methods and commercial reagents (Beckman Coulter Biomedical GmbH, Hamburg, Germany, and BioSystem, Barcelona, Spain) were used to measure CRP and HbA1c. Fibrinogen was measured using the modified coagulometric Clauss method with commercial thrombin reagent (Technoclone, Vienna, Austria) and ESR using the Westergren method.

In the end, using routine lipid profiles, the atherogenic index of plasma (AIP) was calculated, which is defined as the logarithm of plasma triglycerides to HDL-c ratio [29].

### 2.4. Questionnaire

A questionnaire was developed to assess participants’ satisfaction levels regarding their health status, feelings of hunger, and willingness to comply with the diet. The questionnaire was designed using Google Forms, an online survey tool that allows for the easy distribution and collection of responses. The questionnaire consisted of 5 items, each measuring different aspects of satisfaction related to prescribed diet. Each question was formatted using a 10-point Likert scale, where 1 represented the lowest level of satisfaction (e.g., “Very Dissatisfied”) and 10 represented the highest level of satisfaction (e.g., “Very Satisfied”). This scale was chosen to provide a comprehensive range of options for participants to express their levels of satisfaction.

### 2.5. Statistical Analysis

The distribution of data by group was analyzed using the Shapiro–Wilk normality test. Depending on the normality of the data distribution, differences between the groups were analyzed using either the Student’s *t*-test or the Mann–Whitney U-test, and correlations were examined using either the Pearson test or the Spearman test. Using the G-power 3.1.9.7 version, we calculated an n-size of 50, which was sufficient to detect differences, approximately 10%, between baseline weight and post-intervention BW that were shown to have a positive effect on cardiometabolic risk factors, with α = 0.05 and 1 − β = 0.8 [6]. The statistical analysis was performed with the commercial software PASW Statistics Version 18.0 (SPSS Inc., Chicago, IL, USA). The statistical analysis was performed with the commercial software PASW Statistics Version 18.0 (SPSS Inc., Chicago, IL, USA).

## 3. Results

### 3.1. Subjects

Sixty-five participants completed the 14-week study and were included in the analysis. Of these, 34 participants (52.3%) were women (mean age: 39.4 ± 9.8 years; mean BMI: 31.8 ± 9.1 kg/m^2^; mean BF: 41.6 ± 8.3%), and 31 participants (47.5%) were men (mean age: 40.3 ± 9.8 years; mean BMI: 34.1 ± 6.4 kg/m^2^; mean BF: 32.3 ± 6.4%). According to the International Diabetic Federation criteria for MetS (women: WC > 80 cm; men: WC > 94 cm), all women (34/34, 100%) and 97% of men (30/31) among the participants had abdominal obesity; 64% of women (18 of 34) and 36% of men (10 of 31) had elevated triglyceride levels (>1.7 mmol L^−1^); 35% of female participants (12 of 34) and 36% of male participants (11 of 31) had decreased HDL-C (<1.29 in women and <1.03 for men), and 15% women (5 out of 34) and 48% men had raised fasting plasma glucose (>5.6 mmol L^−1^).

The baseline and follow-up results of body composition, anthropometric indices, and biochemical parameters, including lipoprotein profile, glucose levels, and inflammatory parameters, are presented in Table 2, Table 3 and Table 4.

### 3.2. Body Composition and Anthropometric Parameters

**Figure 3:**
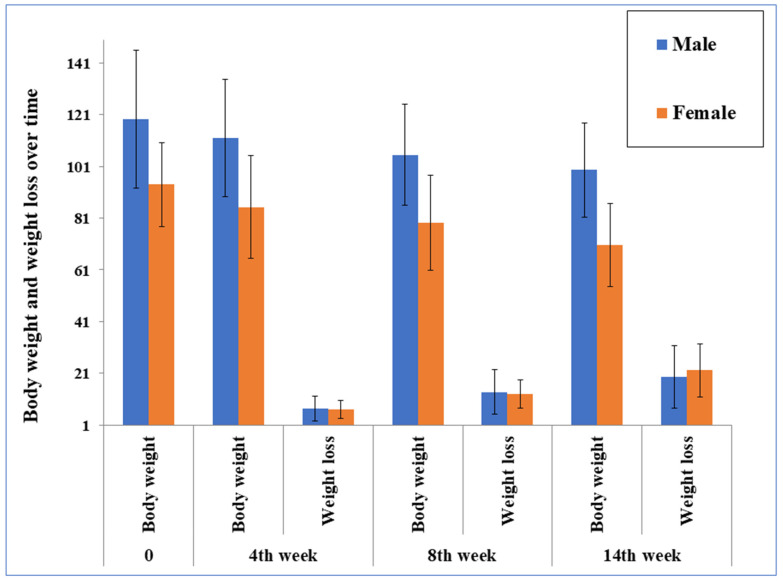
The changes in body weight during the intervention for both sexes.

The results of the 14-week dietary interventions on body composition and anthropometric indices for all participants and separately for men and women and different age subgroups (< > 40 years) are shown in Table 2. A significant decrease in BW (100.1 vs. 83.1 kg, *p* < 0.001), with 64.6% of participants achieving a weight reduction of ≥10%, BF (37.8 vs. 30.6%, *p* < 0.001), BMI (32.9 vs. 26.8 kg m^−2^), and WC (108.5 vs. 96.2 cm, *p* < 0.001) was observed in all participants and the subgroups by gender and age. Similarly, the values of all calculated anthropometric indices were also significantly decreased after the intervention: WHtR (0.62 vs. 0.55, *p* < 0.001); WHR (0.95 vs. 0.93, *p* < 0.001), with the exception of the WHR value for the subgroup of participants over 40 years (0.95 vs. 0.94, *p* = 0.084). In addition, more significant effects of the dietary intervention on BW were found in the subgroup of participants <40 years compared to participants ≥40 years (Δ −19.4 vs. Δ −13.0, *p* = 0.049), as well as on BMI of female participants compared to male participants (Δ −7.2 vs. Δ −5.0, *p* = 0.046). The changes in body weight during the intervention for both sexes are shown in Figure 3. An example of the changes in anthropometric parameters during the intervention for a male participant can be found in Supplementary Materials (Table S3).

### 3.3. Biochemical Parameters and Cardiometabolic Risk Factors

Table 3 shows fasting glucose homeostasis, lipid-related variables, and various indices calculated from specific anthropometric and biochemical parameters and, thus, recognized as predictors of cardiometabolic risks such as MetS.

In summary, significant effects of the 14-week dietary intervention were observed on fasting glucose levels (5.4 vs. 4.9 mmol L^−1^, *p* < 0.001) and glycosylated hemoglobin (HbA1C) (5.3 vs. 5.1%, *p* < 0.001) as markers of glycemic control in all participants and subgroups by age and gender. In addition, significant effects were observed in all lipid-related parameters, with the exception of HDL levels, where a slight but non-significant decrease was observed. Looking at the effects in the subgroups, a significant decrease was only observed in the female subgroup (1.47 vs. 1.33 mmol L^−1^, *p* = 0.013). However, an increase in HDL levels was even observed in the male subgroup, although not significantly (1.17 vs. 1.22 mmol L^−1^, *p* = 0.216), and this effect of the dietary intervention on HDL levels in men was significantly different from that of the female subgroup (Δ 0.05 vs. Δ −0.16, *p* = 0.007).

In addition, the significant effects of the dietary intervention on overweight and obese participants were also assessed using specific indices of body composition and/or adiposity, which integrate anthropometric and biochemical parameters and are, thus, useful and reliable indices for early prediction of cardiometabolic risk. A significant decrease was observed in all predictive indices, except for the AIP score in the female subgroup (−0.04 vs. −0.16, *p* = 0.052), where no significance was reached, although the score was lower compared to baseline. According to the proposed cut-off values for indices that have been shown to be good predictors of cardiometabolic risk in the obese population, such as WtHR (cut-off value = 0.72), VAI (cut-toff value = 1.94), and CMI (cut-off value > 0.84) [18], after the intervention almost twice as many participants with values above the cut-off value for (VAI1 (31/65, 56.9%) and VAI2 (after the intervention) (18/65, 27.7%); CMI1 (28/65, 43.1%) and CMI2 (14/65, 21.5%), a slight decrease in the number of participants above the cut-off value for WtHR: WtHR1 (9/65, 13.8%) and WtHR2 (7/65, 10.8%), where 1 was a value at the entry, and 2 was a value at the end of this study.

Using the proposed cut-off value for AIP (moderate risk: 0.1–0.24 and high risk > 0.24), which has been shown to be one of the strongest markers for predicting coronary heart disease, we also observed that the number of subjects at high risk decreased threefold after the intervention: AIP1 (22/65, 33.8%) and AIP2 (7/65, 10.8%). In addition, there were significant differences in the degree of change between women and men, with the effects being more pronounced in male participants (*p* < 0.028). Among the participants with a cut-off score indicating high risk, 14 were men, and 8 were women. After the intervention, four women and three men were found to be at high risk.

### 3.4. Markers of Inflammation and Markers of Protein Metabolism

Table 4 presents the effects of a specially developed nutritional intervention on inflammation markers and protein metabolism parameters. After the 14-week intervention, CRP levels (4.8 vs. 2.9 mg L^−1^, *p* < 0.001), fibrinogen (3.1 vs. 2.9 g L^−1^, *p* = 0.044), and erythrocyte sedimentation (9.3 vs. 4.1, *p* < 0.001) were significantly decreased in all participants, although the effects on fibrinogen did not reach significance in the male and age subgroups. Effects of the dietary intervention on protein metabolism parameters such as creatinine, blood urea nitrogen (BUN), and uric acid were not observed. However, when considering the effects of the dietary intervention separately in defined subgroups, BUN levels were significantly lower in female participants (4.5 vs. 3.8 mmol L^−1^, *p* = 0.019) and participants aged <40 years (4.9 vs. 4.2 mmol L^−1^, *p* = 0.011).

In addition, a significant decrease in ALT and AST levels as parameters for liver damage and injury was observed in all defined subgroups of participants after the 13-week dietary intervention

### 3.5. Satisfaction Questionnaire

According to the responses from the questionnaire administered at the end of the diet intervention, participants responded with high scores for satisfaction with their health status (9.6 ± 0.7) and diet (9.5 ± 1.4) and willingness to comply with diet (8.6 ± 2.4). Also, participants responded with very low scores for feeling of hunger (3.3 ± 3.0) and intention to withdraw from this study (2.5 ± 2.7) (Supplementary Materials, Figure S1).

## 4. Discussion

Obesity is a major global health problem that is closely associated with an increased risk of many diet-related, non-communicable diseases and mortality [30]. Obesity is characterized by excessive adipose tissue, resulting in dyslipidemia and disturbances in glucose metabolism, which are the major risk factors for the development of atherosclerosis and other cardiometabolic diseases [31]. Over the past two decades, numerous studies have been conducted on the effects of two dietary models, the calorie-restricted diet and the low-CH diet, on the reduction in body weight, especially fat mass, and the reduction in cardiometabolic risk factors.

With regard to the intake of CHs that characterized the days in our study, it is important to note that the intake of CHs is generally based on the established recommended intake of proteins and fats. Furthermore, a reduction in CH intake is always accompanied by a greater or lesser increase in protein intake in the diet, depending on how much additional fat needs to be included in the diet. The results of a meta-analysis from 2018 showed that a CH intake of around 40–70% was associated with a lower risk of death than a lower intake (<40%) or higher intake (>70%) [32]. However, in numerous clinical studies that have been conducted, a low-CH diet in which CH intake was reduced at the expense of increased protein intake (high-protein diet) or a high-fat diet (high-fat diet) in overweight adults had a beneficial effect on adipose tissue, triglyceride levels, serum glucose levels, and the inflammatory factor CRP [31,33,34]. One of the main problems of the very low-carbohydrate diet (<25% of energy intake) on which the keto diet is based is the difficulty of strictly adhering to this diet over a long period of time, as it restricts most foods characteristic of the Western diet, and its positive effect on serum total and LDL cholesterol is unclear [31]. There are certain safety concerns with low-CH diets, particularly diets with a total energy intake of <25%, including potential nutrient deficiencies that can occur when large and important food groups are restricted, the long-term effects of ketosis on bone and kidney health, and the possibility of hyperuricemia [9]. Precisely for these reasons, the dietary intervention in our study was mainly based on a medium CH intake, 40–49% of total energy intake, 5 days per week, while on one day per week, the CH intake was within the standard recommendations (up to 59%). A low-CH diet (<40% of daily energy intake) was recommended only once a week. Our decision to focus on a medium-carbohydrate intake for most of the intervention period was based on the existing literature that highlighted the safety and efficacy of this dietary approach. Namely, our results regarding the effects on lowering lipid parameters and fat mass are consistent with the results of Michalczyk et al. [31], who demonstrated that a low-calorie (20% relative to TDEE) and medium-CH diet (aproxx. 32% of energy intake) had a significant effect on lowering LDL, TG, and body fat. In addition, this diet led to an increase in HDL-C levels in overweight middle-aged men compared to a hypocaloric diet in which the ratio of macronutrients was not altered. Based on the observed beneficial effects in the treatment of obesity, the authors suggested this medium-CH diet as a dietary pattern for obese middle-aged men to effectively reduce adipose tissue while preserving muscle mass. However, in contrast to the study conducted by Michalczyk et al. [31], which lasted only 4 weeks, no statistically significant increase in HDL-C levels was observed in the subgroup of men in our study after 14 weeks of intervention, although the CH content was higher in our study, while the protein content was the same as in the above-mentioned study (about 30% of energy intake). The significant effect of diet on HDL-C levels was observed exclusively in the female subgroup. These results are consistent with the existing literature, which indicates that a low-carbohydrate diet can increase HDL-C levels by an average of 20% [35]. Remarkably, the increase in HDL-C levels was more pronounced in women than men, highlighting gender differences in lipoprotein response to diet [35,36]. The positive effect of diet on HDL-C levels is of great importance, as low HDL-C levels are recognized risk factors for cardiovascular disease and premature atherosclerosis, independent of triglyceride and LDL serum levels [35].

Calorie restriction is the most effective non-pharmacological measure that can lead to a reduction in body weight and prevent chronic metabolic diseases [37]. However, energy-restrictive diets can be problematic as they promote weight loss at the expense of loss of muscle mass and/or a reduction in resting energy expenditure, leading to increased hunger and compensatory motivation to overeat [37,38]. An additional risk factor that may be responsible for an inadequate response to a hypocaloric diet when it is used to reduce body weight is an increased hedonistic response to the sensory properties of foods, such as cravings for fatty, sweet, or salty foods that are normally excluded from the diet [39]. In addition, restrictive diets often trigger adaptive thermogenesis, in which the body reduces its resting metabolic rate and energy expenditure in response to prolonged calorie restriction. This leads to a slowdown in metabolism, which makes it increasingly difficult to maintain weight loss and can lead to weight regain over time [16]. Evidence from clinical studies suggests that increasing the protein content in a hypocaloric diet may promote satiety, probably due to the effect on appetite hormones, but may also lead to an increase in energy expenditure, as proteins have a greater thermic effect compared to fats and CHs [40]. In addition, the use of a hypocaloric diet with a higher protein content compared to the IOM’s stated acceptable macronutrient distribution range (AMDR) for protein (15–25% of total energy intake) [41] and had a more pronounced clinical effect on weight loss and fat mass compared to a diet with a standard amount of protein or a high-CH, low-fat diet [42]. Mateo-Gallego et al. [26] demonstrated that an energy-restricted diet with a high protein content (35% of total energy intake) led to greater fat loss and an improvement in cardiometabolic profile variables compared to a calorie-restricted diet with a lower protein intake (20% of total energy intake) in overweight and obese middle-aged women [26]. Positive effects of a high-protein diet (1.26 to 2.12 g/kg/d) on body composition have also been found in older men (>60 years), even without calorie restriction compared to controls [43]. Finally, adherence to a hypocaloric, high-protein, low-fat diet (32% protein, 12% fat) was more effective in promoting fat loss in both overweight men and women than adherence to a hypoenergetic diet with the same fat content but a protein content equivalent to the AMDR (22% protein, 12% fat) [44]. Consistent with the above research findings, this dietary program used in this study promoted effective loss of body weight, particularly fat mass, with a significant increase in the percentage of skeletal muscle mass in body weight compared to baseline and without the inclusion of planned physical activity. It should be noted that participants followed a diet 5 days a week that included the intake of 1.2–1.4 g/kg/day of protein, corresponding to a daily energy contribution of protein of 25–30% while consuming a standard amount of protein once a week (0.8–1.2 g/kg/day or 15–25% of energy intake). After the intervention, there was no increase in protein metabolic markers such as creatinine, urea, and blood uric nitrogen, which might indicate increased protein catabolism, while in the subgroup of women and people <40, blood uric nitrogen was significantly lower after the intervention.

Diets based on a lower than recommended carbohydrate intake are low in fiber, which largely determines the quality of the carbohydrates. Fiber intake is important for health, especially for gastrointestinal health and function, as well as for improved insulin sensitivity and weight regulation, while low-fiber diets are associated with various metabolic and gastrointestinal disorders [45,46]. Systematic reviews of randomized clinical trials and prospective observational studies in adults have shown that intake of at least 25 g of dietary fiber from fruits, vegetables, and cereals leads to beneficial improvements in obesity and non-communicable diseases with risk factors and is associated with lower risk of all-cause mortality [47]. The threshold of at least 25 g per day was chosen based on observational studies showing a dose–response relationship between dietary fiber intake and reduced risk of various non-communicable diseases and mortality. According to WHO recommendations, for 2000 kcal, the average daily intake of an adult about 25 g of dietary fiber should be consumed, which corresponds to an intake of 0.0125 g of dietary fiber per 1 kcal of energy intake [47]. Since 1 g of fiber has 2 kcal, it can be concluded that fiber should make up at least 2.5% of total energy intake. Since there is evidence that an increase in fiber intake to 50 g per day is likely to extend life expectancy and improve quality of life in the additional years [48], the fiber content in this dietary plan was adjusted to the energy intake, and the proportion of fiber in the total energy intake was kept constant at 5% (Table S1). This ensures that the daily intake of dietary fiber is 10 g and above, depending on the planned daily energy intake. The significant beneficial effects of the dietary regime implemented in this study on lipid parameters (lowering LDL cholesterol and TG and increasing HDL-C) and fasting blood glucose can be explained by planning an adequate intake of dietary fiber even on days when CH intake was <40%. Similarly, in a study conducted by Michalczyk et al., in which they observed the effects of a medium-CH diet in obese middle-aged men, fiber intake was 24.6 ± 2.6 g [31]. Fiber intake may also be responsible for the feeling of satiety reported by the subjects, which, together with a higher protein intake, allowed for better adherence and satisfaction with the prescribed dietary regimen. In addition, promoting the consumption of dietary fiber is crucial in the context of weight loss, as it increases the feeling of satiety, improves fat oxidation, and regulates metabolism. Fiber promotes the growth of beneficial gut bacteria that produce short-chain fatty acids (SCFAs), which help control appetite, increase insulin sensitivity, and reduce inflammation. This positive influence on the gut microbiota contributes to more effective weight control and reduces the metabolic problems associated with obesity [49,50].

With regard to fat intake, the dietary intervention in this study was planned in a way that fat intake followed the change in protein and carbohydrate intake. For example, on days when protein intake was highest and carbohydrate intake was lowest, fats accounted for up to 35% of total energy intake. On medium CH days, fat intake corresponded to AMDR and was in the range of 25–30%, while on maximum CH days, once a week, fat intake could be below AMDR. In studies comparing the effects of a low-carbohydrate diet with a low-fat diet in obese people, the low-carbohydrate diet was found to be more effective in lowering total and LDL-C cholesterol, lowering TG, and increasing HDL levels [51,52]. An important factor influencing the effectiveness of the low-CH diet was the quality of the fats, i.e., a more pronounced effect on the lipid profile was achieved when saturated fats were replaced by monounsaturated and polyunsaturated fats [33,53]. In the diet used in this study, the saturated fat content was <10% of energy intake, which is in line with WHO recommendations [54].

One of the peculiarities of our dietary regime to increase adherence to the diet is the introduction of a day off after three cycles of 3 weeks of hypocaloric diet, on which patients were allowed to consume food ad libitum. The conclusions of this study comparing the success of the 12-month application of the different diet programs (Atkins, Zone, and Ornis) in reducing body weight in obese people were that the effectiveness of the diet depended primarily on adherence to the prescribed diet program and that it was necessary to put more emphasis on strategies to increase adherence in order to increase the effect of weight loss [55]. Studies comparing the effectiveness of calorie-restricted diets and intermittent or alternate-day fasting have also shown that adherence to diets where food was allowed ad libitum on certain days or at certain times was higher [56]. The results of the questionnaire we administered to participants showed very high satisfaction with the diet and a low score for participants’ intention to stop the diet during this study, resulting in high adherence to the diet (Figure S1).

In order to verify the success of the dietary intervention in overweight and obese participants in terms of cardiometabolic risk reduction, the effects of the regimen were also evaluated using specific indices of body composition and/or adiposity that integrated anthropometric and biochemical parameters and, thus, represented useful and reliable indices for the early detection of cardiometabolic risks, particularly MetS. Although there was a significant decrease in all anthropometric parameters and indices, VAI and CMI are reported in the literature as the most informative indices that can predict cardiometabolic risks with high reliability, especially the occurrence of MetS in obese people, particularly in obese women [24]. The VAI is based on two anthropometric parameters (WC and BMI), two biochemical factors (TG and HDL), and the cardiometabolic index (CMI), which integrates the marker for obesity, the WtHR, with the TG/HDL-C ratio. Based on the CMI and VAI values obtained after the 14-week intervention, we can conclude that the number of participants with cardiometabolic risks was halved, with no significant difference between the different subgroups. Furthermore, based on the AIP value obtained after the end of the intervention, the number of participants with a risk for atherosclerosis decreased more than three times. Namely, given the critical role of triglycerides and cholesterol in the pathogenesis of atherosclerotic and cardiovascular diseases, the atherogenic lipoprotein profile of plasma has been identified as a significant risk factor for atherosclerosis [57]. The Atherogenic Index of Plasma, quantitatively defined as the logarithm of the ratio of triglycerides to HDL-C, has emerged as a robust predictor of future cardiovascular events, including coronary artery disease and other cardiovascular diseases [29,58]. Consequently, AIP monitoring can be used together with other parameters to assess the effectiveness of intervention programs to reduce cardiovascular risk [57]. According to the calculated AIP value and the proposed cut-off value for moderate (0.1–0.24) and high risk (>0.24) of cardiovascular disease, there were significant differences between male and female participants in our study, with men having a significantly higher AIP value and diet having a significantly stronger influence on AIP in men. Higher AIP values in the male population were also observed by Dobiásová et al. [59]. In addition, according to the study by Westphal-Nardo et al. [57], AIP proved to be a very valuable tool for predicting cardiometabolic risk in male participants.

Diet adherence is critical to the success of weight loss programs [20]. In this study, several strategies were used to improve diet adherence. These included telephone counseling, a prescribed diet, and ongoing self-monitoring. All of these strategies were found to be effective in promoting long-term diet adherence and successful weight management. Regular contact with healthcare professionals via counseling has been shown to reduce dropout rates and improve diet adherence by providing personalized feedback and addressing challenges that arise [17,20]. In addition, participants in our study received structured meal plans tailored to their needs. Structured diets reduce the cognitive load associated with daily food choices and make it easier for participants to stick to this program. Research shows that clear and structured dietary guidelines are essential for improving adherence, as they reduce confusion and promote adherence [17,20]. In addition, regular follow-up allowed participants to track their progress and receive ongoing feedback. Self-monitoring is widely recognized as one of the most effective strategies to improve adherence to dietary interventions, as it promotes accountability and facilitates timely adjustments [17]. Participants in our study reported their dietary adherence twice a week, which further enhanced their engagement with the intervention and allowed them to receive real-time feedback from medical staff. The literature also emphasizes the importance of tailoring nutritional interventions to individual preferences and dietary needs. This personalization ensures that diets are more sustainable and enjoyable for participants, which is crucial for long-term adherence [60]. In addition, strategies to combat binge eating, such as inducing ketosis through low-carbohydrate days or introducing an ad libitum day, have been shown to suppress cravings and improve long-term adherence to an energy-restricted diet [60].

However, the strategy of alternating carbohydrate intake, coupled with days off and a daily alternating the extent of energy deprivation, was designed to prevent metabolic adaptation. This approach allowed participants to maintain fat oxidation while reducing the compensatory responses that often accompany continuous calorie restriction. By alternating between low, medium, and high carbohydrate intake days, we provided metabolic flexibility, which is essential for maintaining long-term fat loss and avoiding weight plateaus [4,13,14]. While the results of this study provide valuable insights into the potential benefits of a controlled dietary intervention on body composition and cardiometabolic health, several important factors must be considered. First, it should also be noted that the diet used in this study included a structured alternation of carbohydrates, fats, and proteins. The proportion of these macronutrients varied from day to day, with days of minimum, medium, and maximum carbohydrate intake. Therefore, the results observed in this study may be due to the combined effects of changes in carbohydrate, fat, and protein intake rather than carbohydrate intake alone. The relatively small sample size and lack of a control group limit the ability to generalize the results. Without a comparison group, it is difficult to determine whether the observed improvements are specific to the alternative carbohydrate intake or solely due to calorie restriction. Future studies should include randomized control groups adhering to standard dietary interventions to better isolate the effects of this dietary strategy. In addition, the self-selected composition of the participant cohort could lead to selection bias, as individuals who were more motivated to adopt a healthier lifestyle were more likely to volunteer. This may have contributed to the high adherence rates and limited the external validity of the results. Future studies should aim to include a more randomized and representative population to ensure broader applicability of the results. The relatively short duration of the 14-week intervention limits our ability to assess the long-term sustainability of the dietary approach. Although significant improvements in weight loss and cardiometabolic markers were observed in the short term, weight regain and metabolic adjustment are common after dietary interventions. Longer-term follow-up studies are needed to determine whether the benefits observed during the intervention can be maintained over time. One of the greatest strengths of this study was the integration of regular counseling sessions with healthcare professionals and dietitians, which likely played a critical role in promoting adherence and achieving the observed results. The professional support not only helped participants adhere to the dietary protocol but also provided them with personal guidance that strengthened their commitment to this program. However, it is important to realize that constant access to healthcare professionals is not always possible for individuals in the real world, which could limit the generalizability of the results in settings without such support. Nonetheless, this highlights the crucial role that healthcare professionals and dietitians play in helping people achieve and maintain their nutrition and health goals.

## 5. Conclusions

This study showed that a specially designed energy-restricted diet with alternating carbohydrate intake led to a significant reduction in body weight, with more than half of the participants achieving a weight loss of at least 10%. The average weight loss in the entire group was 17%, with a particular increase in the proportion of muscle mass in relation to total body weight. These results underline the effectiveness of the diet in overweight and obese individuals. In addition, the intervention improved important cardiometabolic risk factors, including reductions in fasting blood glucose, LDL cholesterol, and predictive indices of cardiometabolic risk. Gender differences were observed, particularly in HDL-C levels, where the intervention had different effects in men and women, indicating possible gender-specific responses to the diet. The use of alternate days for carbohydrate intake combined with tailored counseling and continuous self-monitoring effectively improved adherence to the diet intervention. This multifaceted approach improved metabolic flexibility and reduced the likelihood of metabolic adaptation, which contributed to sustained fat loss and improved cardiometabolic health. While the dropout rate (65 out of 100 participants completed this study) was comparable to similar studies with intensive monitoring, we believe that the inclusion of structured support mechanisms—such as regular counseling sessions and continuous self-monitoring—helped to reduce the higher dropout rates. While these interventions did not completely prevent participants from dropping out of this study, they helped to ensure that participants stayed with this study for its duration. However, the relatively short duration of this study and the lack of a control group limited the ability to draw long-term conclusions. Future studies should aim to replicate these results with larger, more diverse populations, include control groups, and extend the intervention period to better assess the long-term sustainability and efficacy of this dietary approach.

## 6. Patents

This work has led to the development of a patent titled “Diet Composition for Body Weight Reduction”, with the following details: Inventor: Ana Petrovic; Patent Number: 63407; Application Number: P-2021/0019; Issuing Organization: The Intellectual Property Office of the Republic of Serbia; Year of Issue: 2022; State: Republic of Serbia.

## Figures and Tables

**Table 1:** Macronutrient composition of diet.

	Min Intake	Medium Intake	Max Intake
		Carbohydrates	
%EI	20–35% *	40–49% *	50–65% *
g/kg BW	0.9–1.3	1.9–2.8	2.9–3.6
		Proteins	
%EI	30–40%	25–30%	15–25%
g/kg BW	1.7–1.9	1.2–1.4	0.8–1.2
		Fats	
%EI	30–35%	25–30%	15–20%
g/kg BW	0.6–0.85	0.5–0.6	≤0.5
CH/P ratio in %EI	0.5–1	1.3–1.8	2–2.75
Energy Deficiency	200 kcal up to RMR	200 kcal up to RMR	200 kcal up to RMR

%EI—% contribution to energy intake; BW—body weight; RMR—resting metabolic rate; * fibers max 5% EI.

**Table 2:** Characteristics of the analyzed body composition and anthropometric indexes parameters in the investigated participants (all participants (N = 65), male (N = 31), female (N = 34), younger than 40 years (N = 32), older than 40 years (N = 33)).

Parameters/ Category	All Participants	Change	≤40 Years	Change	> 40 Years	Change	Male	Change	Female	Change
BW, kg										
Value at the baseline	100.1 (93.5–106.8)	−16.2 (−19.4 to −12.3) *	101.3 (90.2–112.4)	−19.4 (−24.9 to 13.9) *;†	98.9 (90.9–107.1)	−13.0 (16.5 to 9.5) *	115.6 (105.9–125.3)	−16.9 (−22.4 to −11.6) *	85.9 (79.6–92.3)	−15.4 (−19.5 to −11.4) *
Value at the end	83.9 (79.1–88.8)		81.9 (74.3–89.4)		85.9 (79.4–92.5)		98.7 (29.8–104.6)		70.5 (66.6–74.4)	
BMI, kg/m^2^										
Value at the baseline	32.9 (30.9–34.9)	−6.1 (−7.6 to −4.5) *	31.9 (29.0–34.7)	−6.0 (−7.9 to 4.2) *	33.9 (29.1–34.9)	−6.3 (−8.7 to −3.9) *	34.1 (31.8–36.5)	−5.0 (−6.6 to 3.6) *;‡	31.9 (28.7–35.1)	−7.2 (−9.7 to 4.7) *
Value at the end	26.8 (25.8–27.8)		25.9 (26.3–28.9)		27.6 (26.3–28.9)		29.1 (27.9–30.3)		24.7 (23.5–25.9)	
WC, cm										
Value at the baseline	108.5 (104.9–112.1)	−12.3 (−14.6 to −10.0) *	107.9 (102.1–113.6)	−13.5 (−17.3 to −9.6) *	109.1 (104.4–113.8)	−11.2 (−14.0 to −8.3) *	111.4 (107.0–115.8)	12.0 (−14.8 to −9.1) *	105.8 (100.1–111.4)	−12.5 (−16.3 to −8.8) *
Value at the end	96.2 (93.9–98.5)		94.4 (91.1–97.6)		97.9 (94.7–101.1)		99.4 (96.6–102.3)		93.2 (89.9–96.5)	
HC, cm										
Value at the baseline	114.8 (111.4–118.1)	−10.9 (−12.9 to −8.9) *	114.6 (109.3–119.9)	−11.6 (−15.0 to −8.2) *	114.9 (110.5–119.3)	−10.2 (−12.6 to −7.8) *	118.0 (112.8–123.0)	−11.4 (−14.6 to −8.2) *	111.8 (107.5–116.1)	−10.4. (−13.1 to −7.7) *
Value at the end	103.9 (101.8–105.9)		103.0 (100.1–105.8)		104.7 (101.6–107.8)		106.5 (103.4–109.7)		101.4 (98.8–103.9)	
BF, %										
Value at the baseline	37.8 (35.3–40.3)	−7.2 (−9.4 to −5.1) *	38.7 (34.3–39.6)	−9.2 (−13.2 to −5.3) *	36.9 (34.3–39.6)	−5.3 (−7.3 to −3.3) *	33.7 (29.9–37.4)	−5.9 (−9.9 to −1.8) **	41.6 (38.7–44.5)	−8.5 (−10.6 to −6.4) *
Value at the end	30.6 (28.9–32.2)		29.5 (27.1–31.8)		31.6 (29.4–33.9)		27.8 (25.9–29.7)		33.1 (30.8–35.5)	
SMM, %										
Value at the baseline	28.1 (26.9–29.3)	2.6 (1.7 to 3.4) *	28.2 (26.3–30.2)	3.0 (1.7 to 4.3) *	28.0 (26.5–29.6)	2.2 (1.0 to 3.4) **	31.2 (29.5–32.8)	2.1 (0.6 to 3.7) **	25.4 (24.2–26.5)	2.9 (2.0 to 3.9) *
Value at the end	30.7 (29.7–31.7)		30.2 (29.7–32.7)		30.2 (28.8–31.6)		33.3 (32.2.−34.4)		28.3 (27.2–29.4)	
Water, %										
Value at the baseline	47.0 (45.4–48.6)	4.2 (2.8 to 5.6) *	48.5 (46.3–50.7)	4.5 (2.4 to 6.4) *	45.6 (43.3–47.9)	3.8 (1.9 to 5.8) *	48.3 (46.2–50.4)	3.4 (1.9 to 4.9) *	45.9 (42.4–48.3)	4.9 (2.6 to 7.2) *
Value at the end	51.2 (50.4–52.1)		53.0 (51.9–54.1)		49.4 (48.6–50.4)		51.7 (50.6–52.9)		50.8 (49.5–52.0)	
WHtR										
Value at the baseline	0.62 (0.59 to 0.64)	−0.07 (−0.08 to −0.06) *	0.61 (0.57 to 0.65)	−0.08 (−0.09 to −0.05) *	0.63 (0.60 to 0.65)	−0.06 (−0.08 to −0.05) *	0.61 (0.58 to 0.63)	−0.07 (−0.08 to −0.05) *	0.63 (0.59 to 0.67)	−0.8 (−0.9 to −0.05) *
Value at the end	0.55 (0.53 to 0.56)		0.53 (0.52 to 0.55)		0.56 (0.54 to 0.58)		0.54 (0.52 to 0.56)		0.55 (0.53 to 0.58)	
WHR										
Value at the baseline	0.95 (0.93 to 0.96)	−0.02 (−0.03 to −0.01) **	0.94 (0.91 to 0.97)	−0.02 (−0.04 to −0.01) **	0.95 (0.93 to 0.97)	−0.01 (−0.03 to −0.001)	0.95 (0.93 to 0.96)	−0.01 (−0.02 to −0.001) ***	0.95 (0.91 to 0.98)	−0.03 (−0.04 to −0.01) **
Value at the end	0.93 (0.91 to 0.94)		0.92 (0.89 to 0.94)		0.94 (0.91 to 0.96)		0.93 (0.92 to 0.95)		0.92 (0.89 to 0.95)	

BW, body weight; BMI, body mass index; WC, waist circumference; HC, hip circumference; BF, body fat; SMM, skeletal muscle mass; WHtR, waist-to-hight ratio; WHR, waist-to-hip ratio; Value is presented as mean and 95% confidence interval (CI); * *p* < 0.001; ** *p* < 0.01; *** *p* < 0.05; statistical difference between younger and older than 40 years: † *p* < 0.05; statistical difference between male and female: ‡ *p* < 0.05.

**Table 3:** Characteristics of the analyzed cardiometabolic parameters and indexes in the investigated participants (all participants (N = 65), male (N = 31), female (N = 34), younger than 40 years (N = 32), older than 40 years (N = 33)).

Parameters/ Category	All Participants	Change	Male	Change	Female	Change	≤40 Years	Change	>40 Years	Change
Glucose, mmol/L										
Value at the baseline	5.4 (5.2 to 5.5)	−0.5 (−0.7 to −0.3) *	5.6 (5.4 to 5.9)	−0.7 (−0.15 to −0.07) *;††	5.1 (4.9 to 5.3)	−0.3 (−0.39 to −0.09) **	5.3 (5.0 to 5.5)	−0.4 (−0.6 to 0.1) **	5.4 (5.2 to 5.6)	−0.5 (−0.8 to −0.3) *
Value at the end	4.9 (4.7 to 5.0)		4.9 (4.8 to 5.1)		4.8 (4.6 to 5.0)		4.9 (4.7 to 5.1)		4.9 (4.7 to 5.1)	
HbA1c, %										
Value at the baseline	5.3 (5.2 to 5.4)	−0.2 (−0.5 to −0.1) **	5.3 (5.2 to 5.5)	−0.2 (−0.4 to −0.1) ***	5.3 (5.2 to 5.4)	−0.3 (−0.4 to −0.03) ***	5.2 (5.1 to 5.4)	−0.1 (−0.3 to −0.002) ***	5.3 (5.2 to 5.5)	−0.2 (−0.4 to −0.1) **
Value at the end	5.1 (4.9 to 5.2)		5.1 (5.0 to 5.2)		5.0 (4.9 to 5.2)		5.1 (4.9 to 5.2)		5.1 (4.9 to 5.2)	
Total cholesterol, mmol/L										
Value at the baseline	5.52 (5.29 to 5.74)	−0.89 (−0.95 to −0.80) *	5.53 (5.25 to 5.81)	−0.82 (−0.89 to −0.76) *	5.51 (5.15 to 5.87)	−0.96 (−1.24 to −0.67) *	5.29 (4.99 to 5.58)	−0.78 (−1.17 to −0.39) *	5.74 (5.41 to 6.08)	−0.99 (−1.30 to −0.68) *
Value at the end	4.63 (4.44 to 4.82)		4.71 (4.38 to 5.04)		4.56 (4.33 to 4.78)		4.51 (4.21 to 4.80)		4.75 (4.49 to 5.01)	
HDL-cholesterol, mmol/L										
Value at the baseline	1.32 (1.23 to 1.43)	−0.04 (−0.05 to −0.03)	1.17 (1.05 to 1.29)	0.05 (0.01 to 0.17) ††	1.47 (1.34 to 1.61)	−0.16 (−0.24 to −0.03) ***	1.34 (1.20 to 1.49)	−0.06 (−0.16 to −0.04)	1.31 (1.18 to 1.45)	−0.04 (−0.14 to 0.07)
Value at the end	1.28 (1.21 to 1.35)		1.22 (1.12 to 1.34)		1.33 (1.24 to 1.42)		1.28 (1.19 to 1.38)		1.27 (1.17 to 1.39)	
LDL-cholesterol, mmol/L										
Value at the baseline	3.38 (3.16 to 3.59)	−0.57 (−0.60 to −0.40) *	3.44 (3.18 to 3.72)	−0.58 (−0.90 to −0.26) **	3.32 (2.98 to 3.66)	−0.57 (−0.86 to −0.27) *	3.23 (2.99 to 3.47)	−0.50 (−0.72 to −0.29) *	3.53 (3.17 to 3.88)	−0.65 (−1.01 to −0.27) **
Value at the end	2.81 (2.65 to 2.96)		2.86 (2.61 to 3.12)		2.75 (2.55 to 2.95)		2.73 (2.53 to 2.92)		2.88 (2.63 to 3.14)	
Triglycerides, mmol/L										
Value at the baseline	2.09 (1.67 to 2.52)	−0.80 (−0.99 to −0.50) *	2.67 (1.91 to 3.43)	−1.06 (−1.67 to 0.45) **	1.56 (1.19 to 1.54)	−0.55 (−0.71 to −0.37) **	1.76 (1.18 to 2.35)	−0.39 (−0.88 to −0.08) ‡	2.41 (1.79 to 3.04)	−1–19 (−1.68 to −0.69) *
Value at the end	1.29 (0.91 to 1.68)		1.61 (0.81 to 2.40)		1.01 (0.85 to 1.18)		1.36 (0.59 to 2.14)		1.22 (1.01 to 1.45)	
AIP										
Value at the baseline	0.11 (0.01 to 0.21)	−0.19 (−0.26 to −0.12) *	0.28 (0.01 to 0.42)	−0.28 (−0.36 to −0.19) *;†	−0.04 (−0.16 to −0.07)	−0.12 (−0.22 to −0.001)	0.03 (−0.11 to 0.16)	−0.14 (−0.26 to −0.03) ***	0.18 (0.04 to 0.33)	−0.23 (−0.33 to −0.14) *
Value at the end	−0.08 (−0.16 to −0.01)		−0.001 (−0.13 to 0.13)		−0.16 (−0.24 to −0.07)		−0.11 (−0.24 to 0.01)		−0.05 (−0.15 to 0.05)	
CMI										
Value at the baseline	1.09 (0.79 to 1.34)	−0.59 (−0.83 to −0.35) *	1.41 (0.87 to 1.96)	−0.85 (−1.31 to −0.40) **;†	0.79 (0.55 to 1.03)	−0.35 (−0.56 to −0.14) **	0.81 (0.57 to 1.05)	−0.37 (−0.58 to −0.17) **	1.36 (0.83 to 1.88)	−0.80 (−1.23 to −0.36) **
Value at the end	0.49 (0.42 to 0.58)		0.56 (0.42 to 0.70)		0.44 (0.35 to 0.54)		0.44 (0.33 to 0.54)		0.56 (0.43 to 0.69)	
VAI										
Value at the baseline			2.5 (1.8 to 3.2)	−1.2 (−1.8 to −0.6) *	3.7 (2.6 to 4.7)	−1.8 (−2.6 to −0.9) *				
Value at the end			1.3 (1.1 to 1.5)		1.9 (1.6 to 2.2)					
LAP										
Value at the baseline			112 (80 to 144)	−71 (−97 to −44) *	76 (54 to 99)	−80 (−110 to −49) **				
Value at the end			41 (32 to 50)		35 (28 to 43)					

HDL, high-density cholesterol; LDL, low-density cholesterol; AIP, atherogenic index of plasma; CMI, cardiometabolic index; VAI, visceral adiposity index; LAP, lipid accumulation product; Value is presented as mean and 95% confidence interval (CI); * *p* < 0.001; ** *p* < 0.01; *** *p* < 0.05; statistical difference between male and female: † *p* < 0.05; †† *p* < 0.01; statistical difference between younger and older than 40 years: ‡ *p* < 0.05.

**Table 4:** Characteristics of the analyzed inflammatory markers and biochemical parameters in the investigated participants (all participants (N = 65), male (N = 31), female (N = 34), younger than 40 years (N = 32), older than 40 years (N = 33)).

Parameters/ Category	All Participants	Change	Male	Change	Female	Change	≤40 Years	Change	>40 Years	Change
CRP, mg/L										
Value at the baseline	4.8 (4.2 to 5.3)	−2.8 (−3.4 to −2.2) *	4.5 (3.7 to 5.4)	−2.4 (−3.2 to −1.6) *	4.9 (4.3 to 5.6)	−3.1 (−3.9 to −2.4) *	5.1 (4.4 to 5.9)	−3.2 (−3.9 to −2.5) *	4.4 (3.6 to 5.2)	−2.5 (−3.3 to 1.6) *
Value at the end	2.0 (1.6 to 2.3)		2.1 (1.6 2.7)		1.8 (1.4 to 2.2)		1.9 (1.5 to 2.4)		1.9 (1.5 to 2.5)	
Fibrinogen, g/L										
Value at the baseline	3.1 (2.9 to 3.3)	−0.2 (−0.4 to −0.01) ***	2.9 (2.7 to 3.2)	0.1 (−0.2 to 0.3) †	3.3 (3.1 to 3.5)	−0.4 (−0.8 to −0.1) **	3.1 (2.8 to 3.3)	−0.3 (−0.6 to −0.1)	3.2 (2.9 to 3.4)	−0.2 (−0.5 to 0.1)
Value at the end	2.9 (2.7 to 3.1)		2.8 (2.7 to 3.3)		2.9 (2.6 to 3.1)		2.8 (2.5 to 3.1)		3.0 (2.7 to 3.3)	
Sedimentation, mm/h										
Value at the baseline	9.3 (8.4 to 10.2)	−5.2 (−6.1 to −4.2) *	9.0 (7.4 to 10.6)	−5.0 (−6.6 to −3.4) *	9.6 (8.5 to 10.7)	−5.3 (−6.4 to −4.1) *	2.3 (8.1 to 10.5)	−5.0 (−6.5 to −3.7) *	9.3 (7.9 to 10.8)	−5.1 (−6.5 to −3.8) *
Value at the end	4.1 (3.8 to 4.6)		4.0 (3.5 to 4.5)		4.3 (3.7 to 4.9)		4.2 (3.5 to 4.9)		4.2 (3.7 to 4.6)	
BUN, mmol/L										
Value at the baseline	4.8 (4.5 to 5.2)	−0.1 (−0.7 to 0.7)	5.2 (4.6 to 5.8)	0.7 (−0.6 to 2.1) †	4.5 (4.1 to 4.9)	−0.7 (−1.2 to −0.1) ***	4.9 (4.5 to 5.3)	−0.7 (−1.2 to 0.2) ***	4.8 (4.2 to 5.4)	0.6 (−0.7 to 1.9)
Value at the end	4.7 (4.2 to 5.4)		5.9 (4.9 to 7.0)		3.8 (3.4 to 4.3)		4.2 (3.7 to 4.7)		5.4 (4.4 to 6.5)	
Creatinine, μmol/L										
Value at the baseline	76.8 (72.6 to 80.9)	−0.5 (−4.7 to 4.7)	88.5 (83.1 to 93.9)	2.0 (−7.5 to 11.6)	66.0 (62.4 to 69.8)	−1.7 (−5.4 to 1.9)	75.1 (68.6 to 81.5)	−1.9 (−6.3 to 2.5)	78.4 (72.7 to 84.1)	1.9 (−6.8 to 10.7)
Value at the end	76.3 (70.9 to 82.6)		90.5 (81.7 to 99.3)		64.3 (59.2 to 69.4)		73.2 (66.0 to 80.3)		80.3 (70.9 to 89.7)	
Uric acid, mmol/L										
Value at the baseline	335 (310 to 361)	−4 (−26 to 18)	391 (356 to 426)	−9 (−47 to 29)	286 (257 to 314)	−1 (−26 to 27)	340 (302 to 378)	−19 (−47 to 9)	332 (296 to 368)	11 (−25 to 46)
Value at the end	331 (312 to 352)		382 (356 to 407)		285 (266 to 307)		321 (289 to 352)		343 (317 to 369)	
Total bilirubin, µmol/L										
Value at the baseline	12.9 (11.1 to 14.9)	1 (−0.7 to to 2.6)	16.0 (12.6 to 19.5)	1.6 (−1.2 to 4.4) †	10.1 (8.6 to 11.7)	0.5 (−1.6 to 2.4)	13.2 (10.4 to 15.9)	2.9 (0.5 to 5.4) ***	12.8 (9.9 to 15.5)	−1.0 (−3.1 to 1.2)
Value at the end	13.9 (11.4 to 16.4)		17.6 (12.9 to 22.4)		10.6 (9.1 to 12.1)		16.1 (11.7 to 20.5)		11.8 (9.4 to 14.3)	
Serum iron, µmol/L										
Value at the baseline	18.5 (16.9 to 20.0)	−0.9 (−2.8 to 0.8)	20.2 (18.0 to 22.3)	−1.5 (−4.7 to 1.8)	16.9 (14.7 to 19.1)	−0.6 (−2.5 to 1.4)	18.3 (16.3 to 20.3)	0.3 (−1.8 to 2.5)	18.6 (16.2 to 21.1)	−2.3 (−5.3 to 0.7)
Value at the end	17.4 (15.9 to 18.9)		18.7 (16.6 to 20.8)		16.3 (14.2 to 18.4)		18.6 (16.4 to 20.8)		16.3 (14.3 to 18.4)	
AST, IU/L										
Value at the baseline	26 (23 to 29)	−7 (−9 to −4) *	29 (24 to 33)	−7 (−11 to −2) **	23 (19 to 28)	−6.6 (−10.7 to −2.5) **	28 (22 to 34)	−10 (−14 to −5) *	24 (21 to 27)	−4 (−7 to −1) ***
Value at the end	19 (18 to 20)		22 (19 to 25)		17 (15 to 19)		18 (16 to 21)		20 (18 to 22)	
ALT, IU/L										
Value at the baseline	35 (28 to 42)	−12 (−18 to −7) *	43 (31 to 54)	−16 (−24 to −6) **	28 (21 to 35)	−9.9 (−17.1 to −2.8) **	40 (27 to 53)	−18 (−28 to −7) **	30 (25 to 36)	−7 (−13 to −2) ***
Value at the end	23 (20 to 25)		27 (22 to 33)		18 (16 to 20)		22 (17 to 28)		23 (20 to 26)	

CRP, C-reactive protein; WBC, white blood cells; BUN, blood urea nitrogen; AST, aspartate aminotranspherase; ALT, alanine aminotranspherase; value is presented as mean and 95% confidence interval (CI); * *p* < 0.001; ** *p* < 0.01; *** *p* < 0.05; statistical difference between male and female: † *p* < 0.05;

## Data Availability

The data presented in this study are available upon request from the corresponding author due to ethical restrictions.

## Supplementary Materials

The following supporting information can be downloaded at https://www.mdpi.com/article/10.3390/nu16203453/s1, Figure S1. Satisfaction questionnaire performed on participants after 14 weeks of dietary intervention; Table S1. Example of a meal plan for 1 cycle for one participant; Table S2. Macronutrient composition of the diet using the example of a participant; Table S3. Examples of changes in a participant’s anthropometric parameters during the dietary intervention.

## References

[1] Aaseth J., Ellefsen S., Alehagen U., Sundfør T.M., Alexander J. (2021). Diets and drugs for weight loss and health in obesity–An update. Biomed. Pharmacother..

[2] NCD Risk Factor Collaboration (NCD-RisC) (2024). Worldwide trends in underweight and obesity from 1990 to 2022: A pooled analysis of 3663 population-representative studies with 222 million children, adolescents, and adults. Lancet.

[3] Schutte S., Esser D., Siebelink E., Michielsen C.J., Daanje M., Matualatupauw J.C., Boshuizen H.C., Mensink M., Afman L.A., Wageningen Belly Fat Study Team (2022). Diverging metabolic effects of 2 energy-restricted diets differing in nutrient quality: A 12-week randomized controlled trial in subjects with abdominal obesity. Am. J. Clin. Nutr..

[4] Cioffi I., Evangelista A., Ponzo V., Ciccone G., Soldati L., Santarpia L., Contaldo F., Pasanisi F., Ghigo E., Bo S. (2018). Intermittent versus continuous energy restriction on weight loss and cardiometabolic outcomes: A systematic review and meta-analysis of randomized controlled trials. J. Transl. Med..

[5] Hołowko J., Michalczyk M.M., Zając A., Czerwińska-Rogowska M., Ryterska K., Banaszczak M., Jakubczyk K., Stachowska E. (2019). Six weeks of calorie restriction improves body composition and lipid profile in obese and overweight former athletes. Nutrients.

[6] Magkos F., Fraterrigo G., Yoshino J., Luecking C., Kirbach K., Kelly S.C., de Las Fuentes L., He S., Okunade A.L., Patterson B.W. (2016). Effects of moderate and subsequent progressive weight loss on metabolic function and adipose tissue biology in humans with obesity. Cell Metab..

[7] Ludwig D.S., Ebbeling C.B. (2018). The carbohydrate-insulin model of obesity: Beyond “calories in, calories out”. JAMA Intern. Med..

[8] Astrup A., Raben A., Geiker N. (2015). The role of higher protein diets in weight control and obesity-related comorbidities. Int. J. Obes..

[9] Pavlidou E., Papadopoulou S.K., Fasoulas A., Mantzorou M., Giaginis C. (2023). Clinical evidence of low-carbohydrate diets against obesity and diabetes mellitus. Metabolites.

[10] Al-Reshed F., Sindhu S., Al Madhoun A., Bahman F., AlSaeed H., Akhter N., Malik M.Z., Alzaid F., Al-Mulla F., Ahmad R. (2023). Low carbohydrate intake correlates with trends of insulin resistance and metabolic acidosis in healthy lean individuals. Front. Public Health.

[11] Hall K.D., Guo J. (2017). Obesity energetics: Body weight regulation and the effects of diet composition. Gastroenterology.

[12] Ma Y., Sun L., Mu Z. (2023). Effects of different weight loss dietary interventions on body mass index and glucose and lipid metabolism in obese patients. Medicine.

[13] Prins P.J., Noakes T.D., Buxton J.D., Welton G.L., Raabe A.S., Scott K.E., Atwell A.D., Haley S.J., Esbenshade N.J., Abraham J. (2023). High fat diet improves metabolic flexibility during progressive exercise to exhaustion (VO2max testing) and during 5 km running time trials. Biol. Sport.

[14] Elaine A.Y., Le N.A., Stein A.D. (2021). Measuring postprandial metabolic flexibility to assess metabolic health and disease. J. Nutr..

[15] Dulloo A.G., Schutz Y. (2015). Adaptive thermogenesis in resistance to obesity therapies: Issues in quantifying thrifty energy expenditure phenotypes in humans. Curr. Obes. Rep..

[16] Anton S.D., Moehl K., Donahoo W.T., Marosi K., Lee S.A., Mainous A.G., Leeuwenburgh C., Mattson M.P. (2018). Flipping the metabolic switch: Understanding and applying the health benefits of fasting. Obesity.

[17] Wang D., Benito P.J., Rubio-Arias J.Á., Ramos-Campo D.J., Rojo-Tirado M.A. (2024). Exploring factors of adherence to weight loss interventions in population with overweight/obesity: An umbrella review. Obes. Rev..

[18] Lee S.A., Sypniewski C., Bensadon B.A., McLaren C., Donahoo W.T., Sibille K.T., Anton S. (2020). Determinants of adherence in time-restricted feeding in older adults: Lessons from a pilot study. Nutrients.

[19] Berthoud H.R., Seeley R.J., Roberts S.B. (2021). Physiology of energy intake in the weight-reduced state. Obesity.

[20] Trujillo-Garrido N., Santi-Cano M.J. (2022). Motivation and limiting factors for adherence to weight loss interventions among patients with obesity in primary care. Nutrients.

[21] Zhang Y., Gu Y.a., Wang N., Zhao Q., Ng N., Wang R., Zhou X., Jiang Y., Wang W., Zhao G. (2019). Association between anthropometric indicators of obesity and cardiovascular risk factors among adults in Shanghai, China. BMC Public Health.

[22] Golabi S., Ajloo S., Maghsoudi F., Adelipour M., Naghashpour M. (2021). Associations between traditional and non-traditional anthropometric indices and cardiometabolic risk factors among inpatients with type 2 diabetes mellitus: A cross-sectional study. J. Int. Med. Res..

[23] Lampignano L., Zupo R., Donghia R., Guerra V., Castellana F., Murro I., Di Noia C., Sardone R., Giannelli G., De Pergola G. (2020). Cross-sectional relationship among different anthropometric parameters and cardio-metabolic risk factors in a cohort of patients with overweight or obesity. PLoS ONE.

[24] Lazzer S., D’Alleva M., Isola M., De Martino M., Caroli D., Bondesan A., Marra A., Sartorio A. (2023). Cardiometabolic index (CMI) and visceral adiposity index (VAI) highlight a higher risk of metabolic syndrome in women with severe obesity. J. Clin. Med..

[25] Petrovic A. (2022). Diet Composition for Body Weight Reduction. R.S. Patent.

[26] Mateo-Gallego R., Marco-Benedí V., Perez-Calahorra S., Bea A.M., Baila-Rueda L., Lamiquiz-Moneo I., de Castro-Orós I., Cenarro A., Civeira F. (2017). Energy-restricted, high-protein diets more effectively impact cardiometabolic profile in overweight and obese women than lower-protein diets. Clin. Nutr..

[27] Lohman T., Roche A., Martorell R. (1991). Anthropometric Standardization Reference Manual.

[28] Heyward V. (2001). ASEP methods recommendation: Body composition assessment. J. Exerc. Physiol..

[29] Choudhary M.K., Eräranta A., Koskela J., Tikkakoski A.J., Nevalainen P.I., Kähönen M., Mustonen J., Pörsti I. (2019). Atherogenic index of plasma is related to arterial stiffness but not to blood pressure in normotensive and never-treated hypertensive subjects. Blood Press..

[30] Mambrini S.P., Menichetti F., Ravella S., Pellizzari M., De Amicis R., Foppiani A., Battezzati A., Bertoli S., Leone A. (2023). Ultra-processed food consumption and incidence of obesity and cardiometabolic risk factors in adults: A systematic review of prospective studies. Nutrients.

[31] Michalczyk M.M., Maszczyk A., Stastny P. (2020). The effects of low-energy moderate-carbohydrate (MCD) and mixed (MixD) diets on serum lipid profiles and body composition in middle-aged men: A randomized controlled parallel-group clinical trial. Int. J. Environ. Res. Public Health.

[32] Seidelmann S.B., Claggett B., Cheng S., Henglin M., Shah A., Steffen L.M., Folsom A.R., Rimm E.B., Willett W.C., Solomon S.D. (2018). Dietary carbohydrate intake and mortality: A prospective cohort study and meta-analysis. Lancet Public Health.

[33] Volek J.S., Gómez A.L., Kraemer W.J. (2000). Fasting lipoprotein and postprandial triacylglycerol responses to a low-carbohydrate diet supplemented with n-3 fatty acids. J. Am. Coll. Nutr..

[34] Nordmann A.J., Nordmann A., Briel M., Keller U., Yancy W.S., Brehm B.J., Bucher H.C. (2006). Effects of low-carbohydrate vs low-fat diets on weight loss and cardiovascular risk factors: A meta-analysis of randomized controlled trials. Arch. Intern. Med..

[35] Eapen D.J., Kalra G.L., Rifai L., Eapen C.A., Merchant N., Khan B.V. (2010). Raising HDL-C in women. Int. J. Women’s Health.

[36] Knopp R.H., Paramsothy P., Retzlaff B.M., Fish B., Walden C., Dowdy A., Tsunehara C., Aikawa K., Cheung M.C. (2005). Gender differences in lipoprotein metabolism and dietary response: Basis in hormonal differences and implications for cardiovascular disease. Curr. Atheroscler. Rep..

[37] Most J., Redman L.M. (2020). Impact of calorie restriction on energy metabolism in humans. Exp. Gerontol..

[38] Beaulieu K., Casanova N., Oustric P., Turicchi J., Gibbons C., Hopkins M., Varady K., Blundell J., Finlayson G. (2020). Matched weight loss through intermittent or continuous energy restriction does not lead to compensatory increases in appetite and eating behavior in a randomized controlled trial in women with overweight and obesity. J. Nutr..

[39] Blundell J.E., Finlayson G. (2004). Is susceptibility to weight gain characterized by homeostatic or hedonic risk factors for overconsumption?. Physiol. Behav..

[40] Pesta D.H., Samuel V.T. (2014). A high-protein diet for reducing body fat: Mechanisms and possible caveats. Nutr. Metab..

[41] Trumbo P., Schlicker S., Yates A.A., Poos M. (2002). Dietary reference intakes for energy, carbohydrate, fiber, fat, fatty acids, cholesterol, protein and amino acids. J. Am. Diet. Assoc..

[42] Galbreath M., Campbell B., La Bounty P., Bunn J., Dove J., Harvey T., Hudson G., Gutierrez J.L., Levers K., Galvan E. (2018). Effects of adherence to a higher protein diet on weight loss, markers of health, and functional capacity in older women participating in a resistance-based exercise program. Nutrients.

[43] Hunter G.R., McCarthy J.P., Bamman M.M. (2004). Effects of resistance training on older adults. Sports Med..

[44] Lee K., Lee J., Bae W., Choi J., Kim H., Cho B. (2009). Efficacy of low-calorie, partial meal replacement diet plans on weight and abdominal fat in obese subjects with metabolic syndrome: A double-blind, randomised controlled trial of two diet plans-one high in protein and one nutritionally balanced. Int. J. Clin. Pract..

[45] Dodevska M.S., Sobajic S.S., Dragicevic V.D., Stankovic I., Ivanovic N.D., Djordjevic B.I. (2021). The impact of diet and fibre fractions on plasma adipocytokine levels in prediabetic adults. Nutrients.

[46] Myhrstad M.C., Tunsjø H., Charnock C., Telle-Hansen V.H. (2020). Dietary fiber, gut microbiota, and metabolic regulation—Current status in human randomized trials. Nutrients.

[47] World Health Organization (2023). Carbohydrate Intake for Adults and Children: WHO Guideline.

[48] O’Keefe S.J. (2019). The association between dietary fibre deficiency and high-income lifestyle-associated diseases: Burkitt’s hypothesis revisited. Lancet Gastroenterol. Hepatol..

[49] Djordjevic B., Ivanovic N. (2022). Precise Nutrition and Metabolic Syndrome, Remodeling the Microbiome with Polyphenols, Probiotics, and Postbiotics. Advances in Precision Nutrition, Personalization and Healthy Aging.

[50] Petrovičova O.D., Djuričić I., Ivanović N., Dabetić N., Dodevska M., Ilić T. (2024). Dietary interventions in obesity: A narrative review. Arch. Pharm..

[51] Brinkworth G.D., Noakes M., Buckley J.D., Keogh J.B., Clifton P.M. (2009). Long-term effects of a very-low-carbohydrate weight loss diet compared with an isocaloric low-fat diet after 12 mo. Am. J. Clin. Nutr..

[52] Sharman M.J., Gómez A.L., Kraemer W.J., Volek J.S. (2004). Very low-carbohydrate and low-fat diets affect fasting lipids and postprandial lipemia differently in overweight men. J. Nutr..

[53] Dodevska M., Kukic Markovic J., Sofrenic I., Tesevic V., Jankovic M., Djordjevic B., Ivanovic N.D. (2022). Similarities and differences in the nutritional composition of nuts and seeds in Serbia. Front. Nutr..

[54] WHO (2023). Saturated Fatty Acid and Trans-Fatty Acid Intake for Adults and Children: WHO Guideline.

[55] Alhassan S., Kim S., Bersamin A., King A., Gardner C. (2008). Dietary adherence and weight loss success among overweight women: Results from the A TO Z weight loss study. Int. J. Obes..

[56] Kroeger C.M., Trepanowski J.F., Klempel M.C., Barnosky A., Bhutani S., Gabel K., Varady K.A. (2018). Eating behavior traits of successful weight losers during 12 months of alternate-day fasting: An exploratory analysis of a randomized controlled trial. Nutr. Health.

[57] Westphal-Nardo G., Chaput J.-P., Faúndez-Casanova C., Fernandes C.A.M., de Andrade Gonçalves E.C., Utrila R.T., Oltramari K., Grizzo F.M.F., Nardo-Junior N. (2023). Exploring new tools for risk classification among adults with several degrees of obesity. Int. J. Environ. Res. Public Health.

[58] Li Y., Feng Y., Li S., Ma Y., Lin J., Wan J., Zhao M. (2023). The atherogenic index of plasma (AIP) is a predictor for the severity of coronary artery disease. Front. Cardiovasc. Med..

[59] Dobiasova M. (2006). AIP--Atherogenic index of plasma as a significant predictor of cardiovascular risk: From research to practice. Vnitr. Lek..

[60] Gibson A.A., Sainsbury A. (2017). Strategies to improve adherence to dietary weight loss interventions in research and real-world settings. Behav. Sci..

