# Supplementary material for: Effects of Specially Designed Energy-Restricted Diet on Anthropometric Parameters and Cardiometabolic Risk in Overweight and Obese Adults: Pilot Study
Source: Nutrients. 2024 Oct 11;16(20):3453. doi: 10.3390/nu16203453 (PMC11510625; doi:10.3390/nu16203453)
Supplement: Supplementary file 1 [file nutrients-16-03453-s001.zip › Supplement files R2/Supplement Table S1.pdf]

Table S1. Example of a meal plan for 1 cycle for one participant

	Breakfast (in the first hour after waking up)	***Snack (3:45-4h after previously finished meal)***	***Lunch (3:45-4h after previously finished meal)***	***Dinner (3:45-4h after previously finished meal)***	***TOTAL INTAKE***
Medium	*Polenta 350g + fat - free yogurt (*Balance) 500g + 2 spoons of ground flax*	*Fruit salad 650g (apple, kiwi, pear)*	*Grilled turkey or chicken 400g + 250g grilled peppers + cabbage salad 350g + spoon of ground flax*	*Salad with eggs and grilled zucchini according to the recipe*	
INTAKE	***TE 847.7 kcal; CH 136.3g; F18.5 g; P 29.5 g; Fibers 24.53g***	***TE 332 kcal; CH 97.3g; F1.8g; P 4.19g; Fibers 18.47g***	***TE 1083.56 kcal; CH 49.6g; F 42.23g; P 98.65g; Fibers 14.4g***	***TE 724.4kcal; CH 48.49g; F 21.36g; P 57.9g; Fibers 14.9g***	***TE 2987.66 kcal; CH 331.78g; F 83.89g; P 190.24 g; Fibers 72.3g***
Medium	*Fruit mix with 6 spoons of oatmeal + red fruit + almonds (according to the recipe)*	*5 kiwis + 10 almonds*	*Green beans with chicken and vegetables 550g + cabbage salad minimum 200g + 2 spoon of ground flax*	*Steak salad according to the recipe (250g of steak)*	
INTAKE	***TE 834 kcal; CH 142.83g; F13.8 g; P 25.3g; Fibers 23.68g***	***TE 429 kcal; CH 86.8 g; F 7.4g; P 7.3 g; Fibers 10.56g***	***TE 789 kcal; CH 34.8 g; F 32.33g; P 66.78g; Fibers 25.62g***	***TE 663.93 kcal; CH 16.6 g; F 31.87 g; P 72.4 g; Fibers 8.18g***	***TE 2715.93 kcal; CH 281.03g; F 85.4g; P 171.78g; Fibers 68.04g***
Maximum	*Cheese spread (according to the recipe) 400g + 5 slices of integral bread + salad (according to the recipe) minimum 200g*	*Beetroot juice according to the recipe 700ml*	*Green beans with chicken and vegetables 550g + cabbage salad minimum 200g + 2 spoon of ground flax*	*Sea bream salad according to the recipe (250g of fish)*	
INTAKE	***TE 800.35 kcal; CH 171.8g; F 5.09 g; P 28.46 g; Fibers 20.2g***	***TE 739.15 kcal; CH 175.2 g; F 13.31g; P 10.3g; Fibers 18.64g***	***TE 789 kcal; CH 33.1g; F 30.33g; P 57.5g; Fibers 25.62g***	***TE 704.68 kcal; CH 36.3g; F 18g; P 56.9 g; Fibers 12.74g***	***TE 3033.18 kcal; CH 416.40g; F 66.73g; P 153.16g; Fibers 77.2g***
Minimum	*Oatmeal 4 tablespoons + fat-free yogurt (*Balance) 500 ml + red berries 100g + 15 almonds*	*3 apples + cinnamon*	*Chicken white meat with vegetables, mustard and sesame + cabbage salad at least 200 g*	*Vegetable potage according to the recipe 300ml + young cheese 250g + salad minumum 200g*	
INTAKE	***TE 734.61 kcal; CH 80.71 g; F16.3 g; P 26.17 g; Fibers 21.22g***	***TE 343.09 kcal; CH 56.11g; F 0 g; P 0 g; Fibers 16g***	***TE 1035.07 kcal; CH 16.2 g; F 58.07 g; P 143.7g; Fibers 11.8g***	***TE 439.81 kcal; CH 18g; F 26.6 g; P 39.44g; Fibers 12.24g***	***TE 2552.58 kcal; CH 171.02g; F 100.97g; P 209.31 g; Fibers 61.26g***
Medium	*3 integral sandwiches with 300g prosciutto according to the recipe + salad (according to the recipe) minimum 200g*	*Red berries 500g*	*Moussaka with zucchini according to the recipe 550g + mix of green salads minimum 200g*	*Tuna salad according to the recipe (160g of fish)*	
INTAKE	***TE 1142 kcal; CH 97.11g; F 31.3 g; P 99.3g; Fibers 21.4 g***	***TE 264 kcal; CH 58.5g; F 3g; P 4.5 g; Fibers 21.4g***	***TE 853.3 kcal; CH 134g; F 23.38 g; P 43.5g; Fibers 16.8g***	***TE 552.93 kcal; CH 21.14 g; F 20.75g; P 31.28 g; Fibers 14.93g***	***TE 2812.23 kcal; CH 310.75g; F 78.43g; P 178.58g; Fibers 74.53g***
Medium	*Omelette 2 eggs + 7 egg whites + 100g of spinach + 3 slices of integral bread + salad (according to the recipe) minimum 200g*	*Fruit salad 650g (apple, kiwi, orange)*	*Moussaka with zucchini and meat, according to the recipe 450g + mix of green salads minimum 200g*	*Vegetable burger according to the recipe 300g + salad*	
INTAKE	***TE 823.55kcal; CH 67.89 g; F22.56g; P 60.74g; Fibers 14.6g***	***TE 432 kcal; CH 57.3g; F1.8g; P 4.19g; Fibers 18.47g***	***TE 697.21kcal; CH 109.64g; F 27.49g; P 92.35g; Fibers 13.75g***	***TE 649.24 kcal; CH 27.32 g; F 29.1g; P 18.36g; Fibers 14.33g***	***TE 2602 kcal; CH 262.15g; F 80.95g; P 175.64 g; Fibers 61.15g***
Medium	*Muffins with zucchini according to the recipe 5 pcs + salad (according to the recipe) minimum 200g*	*3 apples + 10 almonds + cinnamon*	*Sea bream 500g + zucchini puree according to the recipe 200g + salad minimum 2*	*Caesar salad according to the recipe*	
INTAKE	***TE 825.55 kcal; CH 158.93 g; F 23.48g; P 23.4g; Fibers 17.2g***	***TE 435 kcal; CH 98g; F 7.4 g; P 7.3 g; Fibers 18g***	***TE 1.074 kcal; CH 41.5g; F 26.92g; P 93.87g; Fibers 23.33g***	***TE 589.33 kcal; CH 26.85g; F 24.39 g; P 58.9; Fibers 16.03g***	***TE 2923.88 kcal; CH 325.28.g; F 82.19g; P 183.47g; Fibers 74.56g***

TE - Total Energy; CH-carbohydrates; F-Fats; P-Proteins;

***All dishes are prepared by the respondent according to the recipe that is delivered to him with the menu**

****The subject includes squeezed orange juice on days when he has a drop in energy**
