# Supplementary material for: Effects of Specially Designed Energy-Restricted Diet on Anthropometric Parameters and Cardiometabolic Risk in Overweight and Obese Adults: Pilot Study
Source: Nutrients. 2024 Oct 11;16(20):3453. doi: 10.3390/nu16203453 (PMC11510625; doi:10.3390/nu16203453)
Supplement: Supplementary file 1 [file nutrients-16-03453-s001.zip › Supplement files R2/Supplement Table S2.pdf]

Table S2. Macronutrient composition of the diet using the example of a participant

	Reduction intake minimum ***3,200 kcal***	Reduction intake maximum ***2,500.00***	
Basal metabolism (BMR) kcal	Body Weight (kg)	Height (cm)	Total daily energy consumption (TDEE) kcal
2.478,00	***127,9***	***175***	***3.407,00***

Male, 34 years	
Height (BH) 175 cm	
Body Weight (BW) 127.9 kg	
BMI 41.76 kg/m2	

DAY	***CYCLE***	***∑ (g)***	***CH (g)***	***CH (kcal) 4kcal/g***	***CH (%EI)***	***of which sugars (g)***	***Fats (g)***	***Fats (kcal) 9kcal/g***	***Fats (%EI)***	***of which unsaturated (g)***	***of which saturated (g)***	***Proteins (g)***	***Proteins (kcal) 4kcal/g***	***Proteins (%EI)***	***Fibers (g)***	***Fibers (kcal)*** 2kcal/g	***Fibers (%EI)***	***Total Energy (kcal)***
**1**	medium intake	678,20	331,78	1.327,10	***44,4***	***75,0***	83,89	755,00	***25,3***	***54,0***	***17,0***	190,24	760,96	***25,5***	72,30	144,60	***4,8***	***2.987,66***
**2**	medium intake	606,25	281,03	1.124,12	***41,4***	***68,8***	85,40	768,61	***28,3***	***32,0***	***8,1***	171,78	687,13	***25,3***	68,04	136,07	***5,0***	***2.715,93***
**3**	maximum intake	713,48	416,40	1.665,60	***54,9***	***76,2***	66,73	600,57	***19,8***	***29,8***	***7,3***	153,16	612,62	***20,2***	77,20	154,39	***5,1***	***3.033,18***
**4**	minimum intake	542,56	171,02	684,09	***26,8***	***52,4***	100,97	908,72	***35,6***	***68,9***	***21,7***	209,31	837,25	***32,8***	61,26	122,52	***4,8***	***2.552,58***
**5**	medium intake	642,28	310,75	1.243,00	***44,2***	***69,7***	78,43	705,87	***25,1***	***49,3***	***8,7***	178,58	714,31	***25,4***	74,53	149,05	***5,3***	***2.812,23***
**6**	medium intake	579,88	262,15	1.048,61	***40,3***	***62,0***	80,95	728,56	***28,0***	***50,4***	***8,5***	175,64	702,54	***27,0***	61,15	122,29	***4,7***	***2.602,00***
**7**	medium intake	665,51	325,28	1.301,13	***44,5***	***73,3***	82,19	739,74	***25,3***	***52,0***	***15,6***	183,47	733,89	***25,1***	74,56	149,12	***5,1***	***2.923,88***
