# Supplementary material for: Effects of Specially Designed Energy-Restricted Diet on Anthropometric Parameters and Cardiometabolic Risk in Overweight and Obese Adults: Pilot Study
Source: Nutrients. 2024 Oct 11;16(20):3453. doi: 10.3390/nu16203453 (PMC11510625; doi:10.3390/nu16203453)
Supplement: Supplementary file 1 [file nutrients-16-03453-s001.zip › Supplement files R2/Supplement Table S3.pdf]

**Table S3. Examples of changes in a participant's antropometric parameters during the dietary inte**

| | | I week | II week | IV week | After one cycle | | 14 week |
|---|---|---|---|---|---|---|---|
| | **0th day 15.08.2023.** | **8th day 22.08.2023** | **15th day 29.08.2023.** | **31st day 14.09.2023.** | ***TOTAL*** | ***Results %*** | **98th day 27.11.2023.** |
| ***HEIGHT / cm*** | 175 | 175 | 175 | 175 | / | / | **175** |
| ***WEIGHT / kg*** | 127,9 | 122 | 118,4 | 114,7 | ***-13,20*** | ***-10,32%*** | **97** |
| ***BMI*** | 41,76 | 39,84 | 38,66 | 37,45 | ***-4,31*** | ***-10,32%*** | **31,67** |
| ***% FAT*** | 37,2 | 37,9 | 37,1 | 34,5 | ***-2,70*** | ***-7,26%*** | **25,9** |
| ***% VISCERAL FAT*** | 21 | 20 | 20 | 18 | ***-3,00*** | ***-14,29%*** | **13** |
| ***% MUSCLE MASS*** | 29,3 | 29,1 | 29,5 | 30,9 | ***1,60*** | ***5,46%*** | **34,6** |
| ***% WATER*** | 44,4 | 45,2 | 45,6 | 46,2 | ***1,80*** | ***4,05%*** | **49,4** |
| ***Waist Circumference*** | ***125*** | ***120*** | ***116*** | ***114*** | ***-11,00*** | ***-8,80%*** | **94** |

**ervention**

| After 14 cycles | |
|---|---|
| ***TOTAL*** | ***Results %*** |
| */* | */* |
| ***-30,90*** | ***-24,16%*** |
| ***-10,09*** | ***-24,16%*** |
| ***-11,30*** | ***-30,38%*** |
| ***-8,00*** | ***-38,10%*** |
| ***5,30*** | ***18,09%*** |
| ***5,00*** | ***11,26%*** |
| ***-31,00*** | ***-24,80%*** |
