# Supplementary figures and images for: Effects of Specially Designed Energy-Restricted Diet on Anthropometric Parameters and Cardiometabolic Risk in Overweight and Obese Adults: Pilot Study
Source: Nutrients. 2024 Oct 11;16(20):3453. doi: 10.3390/nu16203453 (PMC11510625; doi:10.3390/nu16203453)

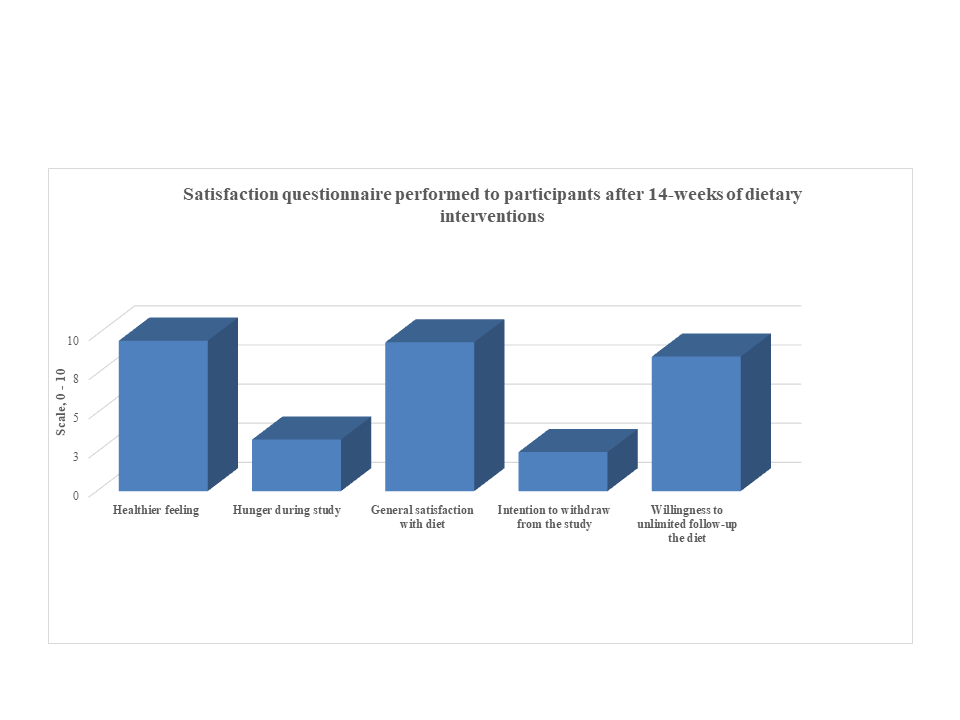

Supplement: Supplementary file 1 [file nutrients-16-03453-s001.zip › Supplement files R2/Supplement Figure S1.tif]
